# Syphilis in the Negro, as Differing from Syphilis in the White Race

**Published:** 1878-11-20

**Authors:** 


### SYPHILIS IN THE NEGRO, AS DIFFERING FROM SYPHILIS IN THE WHITE RACE.

---

Dr. Wm. Powell, of Grenada, Miss., contributes the following paper to the *Transactions of the Mississippi State Medical Association*, 1878:

In preparing a report on the subject assigned to me at our last annual meeting, "Syphilis in the Negro, as differing from Syphilis in the White Race," I have been unable to find anything in reference to it in print, but have been assisted by information kindly furnished by several members of this Association, and will give the result in a condensed form.

The two races, white and black, seem to be equally susceptible to the influence of the syphilitic virus; the primary chancre in its different stages, presenting very much the same appearance. Secondary symptoms are not as frequent in the negro as in the white, occasionally buboes form and in some cases cutaneous eruptions, erythema of the fauces, and mucous tubercles about the privates. Tertiary symptoms, such as so often affect the cutaneous, osseous and nervous system of the white, rarely, if ever, occur in the negro.

Inherited syphilis occurs very frequently in the negro, and is the cause of the greater part of the fatality from this disease. Abortion is often produced, the foetus becomes affected, dies and is expelled; in other cases, the child is born alive, at full term, with syphilitic eruptions, or they come on in a short time, say within six months; by far the greater number of these cases die; according to my observation, very few reach two years of age.

The disease is far more amenable to treatment in the negro, than in the white, the cases yield readily, and the cures are more permanent and satisfactory.

Dr. B. F. Kittrell, of Black Hawk, has furnished a case of a negro man and his wife, both of whom he treated about ten years ago, for primary syphilis. The cases both yielded very readily to treatment; the woman miscarried once or twice, afterward gave birth to syphilitic children at full term. The disease yielded readily to treatment in these children. Her last child did not present any evidence of disease, showing the parents had lost all taint.

Dr. R. S. Riuggold, of Grenada, has furnished a case of a negro man, to whom he was called several months since; on examination, he found a true syphilitic chancre on the body of the penis, and a large bubo in each inguinal region, both of which discharged pus freely on being lanced; he prescribed for the case constitutional remedies and local applications; in about four or five weeks, he met his patient on the street, who reported himself well; on inquiry, the Doctor learned the case had recovered under the use of slippery elm poultices to the buboes, and red oak ooze to the chancre; no constitutional remedy had been taken.

From what I can see and learn, the disease is very much on the increase in the negro population since the day of freedom to the race. This would be readily inferred from the free communication that exists among them, and particularly when we take into consideration the habits of the race, the fact of promiscuous intercourse, want of cleanliness, and of all hygienic regulations.

Comparatively few of the cases are treated by competent physicians; many are treated by quacks of both races, with various nostrums, herbs, roots, etc., and, no doubt, many are not treated at all. We are forced to the conclusion that the system of the African has the power of resisting the influence of the syphilitic virus, of greatly modifying its effects, and of gradually eliminating it from the system; and this probably in proportion to the purity of the African blood—the mulatto suffering more than the negro. But for this provision of nature, the whole negro race would be swept from the face of the earth in a comparatively short time, by this disease alone.

Controversy has been going on for some time, relative to the duality of the syphilitic virus. It has occurred to me that this question is fully solved by the fact that the virus is modified, in passing through the system of the negro. Syphilis imparted from white to white, continues syphilis indefinitely, producing chancre, followed by its train of secondary and tertiary symptoms, imbuing the whole system of the white. Syphilis imparted from the white to the negro, being modified, becomes syphiloid, producing chancroid, rarely followed by secondary and tertiary symptoms, gradually wears out and disappears from the system; and if imparted from the negro to the white, continues to produce the modified form of disease.—*Va. Med. Monthly*, August, 1878.

